# Multifunctional Se–Cu bimetallic nanoparticles from marine *Bacillus licheniformis*: targeting oxidative stress, inflammation, and microbial biofilms

**DOI:** 10.1039/d5ra08848h

**Published:** 2026-02-03

**Authors:** Anes A. Al-Sharqi, Mohamed E. Eissa, Dareen Alyousfi, Ahmed Eid Alharbi, Ibrahim M. Ibrahim, Sozan M. Abdelkhalig, Faisal Miqad K. Albaqami, Ahmed M. Eldesoky, Ahmad A. Sherbini, Tarek A. Yousef, Mohamed N. Goda, Ahmed Ghareeb

**Affiliations:** a Photonics Unit, Institute of Laser for Postgraduate Studies, University of Baghdad Al-Jadiriah, P.O.Box 47314 Baghdad Iraq anes@ilps.uobaghdad.edu.iq; b College of Science, Chemistry Department, Imam Mohammad Ibn Saud Islamic University (IMSIU) Riyadh 11623 Saudi Arabia miissa@imamu.edu.sa tayousef@imamu.edu.sa mnibrahim@imamu.edu.sa; c Department of Clinical Biochemistry, Faculty of Medicine, King Abdulaziz University 21589 Jeddah Saudi Arabia dalyousfi@kau.edu.sa; d Department of Clinical Laboratory Sciences, College of Applied Medical Sciences in Yanbu, Taibah University Saudi Arabia aeharbi@taibahu.edu.sa; e Department of Clinical Pharmacology, Faculty of Medicine, King Abdulaziz University Jeddah 21589 Saudi Arabia imibrahim1@kau.edu.sa asherbini@kau.edu.sa; f Department of Basic Medical Sciences, College of Medicine, AlMaarefa University Diriyah 13713 Riyadh Saudi Arabia sfadl@um.edu.sa; g Research Center, Deanship of Scientific Research and Post-Graduate Studies, AlMaarefa University Diriyah 13713 Riyadh Saudi Arabia sfadl@um.edu.sa; h Biology Department, Faculty of Science, Islamic University of Madinah Madinah 42351 Saudi Arabia falbaqami@iu.edu.sa; i Department of Chemistry, University College in Al-Qunfudhah, Umm Al-Qura University Al-Qunfudhah 21912 Saudi Arabia amahmed@uqu.edu.sa; j Botany and Microbiology Department, Faculty of Science, Suez Canal University Ismailia 41522 Egypt aghareeb@science.suez.edu.eg

## Abstract

Selenium–copper bimetallic nanoparticles (Se–Cu BMNPs) were synthesized using metabolic extracts from the marine bacterium *Bacillus licheniformis* LHG166 isolated from the Red Sea. UV-Vis spectroscopy showed maximum absorption at 208 nm. FT-IR analysis revealed bacterial proteins and polysaccharides from the bacterial extract as reducing and capping agents, showing an Amide I shift to 1646.28 cm^−1^ and new Cu–O/Se–O stretching at 470.34 cm^−1^. XRD patterns confirmed the presence of both orthorhombic and cubic phases of CuSe, with an average crystallite size of 27.2 nm. TEM showed spherical morphologies of 20–120 nm diameter. EDX confirmed Cu : Se atomic ratio near 1 : 1 (7.1 at% Cu, 7.5 at% Se). DLS measured the hydrodynamic diameter of 84 nm (PDI 0.26) with a zeta potential of −24.11 mV. Antioxidant testing showed DPPH scavenging up to 96.1% at maximum concentration with an IC_50_ of 4.1 µg mL^−1^*vs.* ascorbic acid's 3.1 µg mL^−1^, and ABTS scavenging reached 94.6% with an IC_50_ of 10.73 µg mL^−1^ compared to 2.55 µg mL^−1^ for ascorbic acid. Anti-inflammatory assessment demonstrated COX-1 inhibition up to 97.3% (IC_50_ = 7.05 µg mL^−1^) and COX-2 inhibition reaching 95.3% (IC_50_ = 12.11 µg mL^−1^) *vs.* celecoxib's IC_50_ values of 5.93 and 4.51 µg mL^−1^, respectively. Antimicrobial screening *via* agar well diffusion showed inhibition zones of 28 mm for *B. subtilis*, 24 mm for *E. faecalis*, and 27 mm for *C. albicans*. Broth microdilution revealed MIC values ranging from 15.62 µg mL^−1^ (*B. subtilis*, *C. albicans*, *C. tropicalis*) to 125 µg mL^−1^ (*S. aureus*), with MBC/MFC values between 15.62-250 µg mL^−1^, yielding ratios of 1.0–4.0, indicating bactericidal activity. Gram-negative bacteria required 31.25–62.5 µg mL^−1^ for inhibition and 62.5–125 µg mL^−1^ for complete killing, while *A. niger* showed complete resistance. Biofilm inhibition through microtitre plate assays demonstrated concentration-dependent effects, with 75% MBC achieving over 90% inhibition for most organisms (*C. albicans* 96.09%, *B. subtilis* 93.76%, *E. coli* 91.59%), though *S. aureus* required higher concentrations (84.33% at 75% MBC). These results demonstrated that marine bacterial metabolites produce biocompatible Se–Cu BMNPs with potent antioxidant, anti-inflammatory, antimicrobial, and antibiofilm properties suitable for biomedical applications.

## Abstract 2

Selenium–copper bimetallic nanoparticles (Se–Cu BMNPs) were synthesized using metabolic extracts from the marine bacterium *Bacillus licheniformis* LHG166 isolated from the Red Sea.

## Graphical abstract

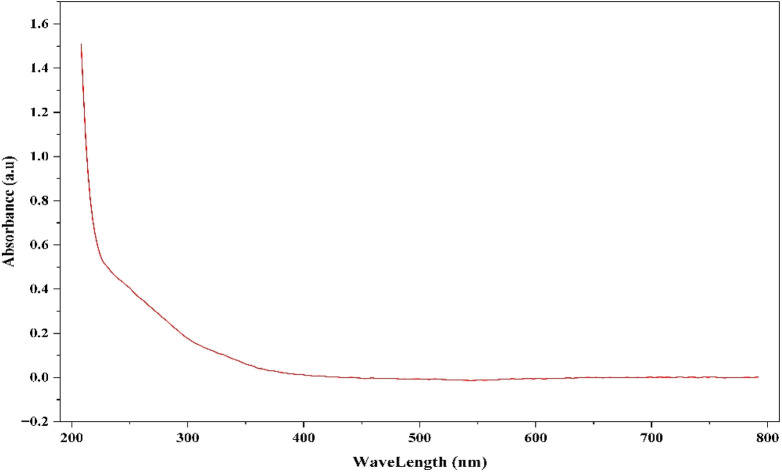

## Introduction

Metal and metalloid nanoparticles serve biomedicine, electronics, and environmental remediation applications, with selenium–copper (Se–Cu) nanoparticles demonstrating notable physicochemical properties and biological activities.^1^ Chemical synthesis requires harsh conditions, toxic reagents, and complex purification steps that reduce environmental sustainability and biocompatibility.^2^ Such syntheses produce single-phase, well-crystalline NPs with defined lattice structures and tight doping control, but create bare inorganic surfaces needing modification for biomedical safety.^3^

Bacteria synthesize metal and metalloid nanoparticles through both intracellular and extracellular pathways by reducing ionic precursors through enzymatic and non-enzymatic metabolites, while capping/stabilizing particles with proteins, exopolysaccharides and biomolecules.^4^ Bacterial cell-free extracts, secreted enzymes, proteins and exopolysaccharides function as dual-purpose agents, serving as reducing agents (electron donors or redox enzymes) and capping/stabilizing agents during nanoparticle synthesis.^5^ Conjugation with EPS or chitosan modifies SeNPs' shape, stability and hemocompatibility through natural stabilization that improves biocompatibility.^6^

Microbial routes operate under mild conditions (ambient temperature, aqueous media) with intrinsic functionalization, without toxic reagents needed in chemical syntheses.^7^ Bacterial metabolite biosynthesis uses different reactants, conditions, and product surface chemistry than conventional chemical routes, creating NPs with distinct safety and application properties.^8^ Microbial methods skip strong reductants, organic solvents and stabilizers from chemical coprecipitation or thermal syntheses, reducing environmental and handling hazards.^9^ Bacterial-derived coatings and controlled intracellular *versus* extracellular formation modify size, morphology and surface chemistry, affecting antimicrobial and anticancer potency through design approaches for specific applications.^10^ Microbial approaches create biocompatible nanomaterials with built-in functionality without additional modification steps, yet match comparable size control.^11^

Bacteria synthesize Se–Cu NPs through enzyme- and metabolite-mediated reduction and capping, creating protein/polysaccharide-coated particles with antimicrobial, anticancer, antioxidant and wound-healing activities.^12,13^ Microbes accumulate selenite or copper intracellularly and reduce them enzymatically to elemental Se(0) or Cu(i/ii) elements, with SeNPs released *via* cell lysis or secretion within 24–72 hours in probiotic and lactic bacteria and fungi.^14–16^ Extracellular synthesis uses selenite-reducing bacteria like *Pantoea agglomerans* with chemically reduced Cu^+^ to catalyze Se^2−^ formation and Cu_2_Se nanoparticle formation, producing protein-wrapped Cu_2_Se nanospheres ∼100 nm in diameter in the extracellular medium.^17^ Microbial NPs carry protein, lipid or polysaccharide coatings from producing organisms, improving aqueous stability and shifting zeta potential to stable ranges (−28 to −35 mV for biogenic CuO and Se NPs) with reduced acute toxicity *versus* metal salts or ionic forms. Pathways include enzymatic selenite reduction, electron transfer by reduced copper species, and metabolite-driven nucleation in coupled biological-chemical schemes for Cu–Se formation.^18^

While monometallic nanoparticles have shown promise, bimetallic systems offer enhanced properties through synergistic effects.^19^ Cu–Se bimetallic NPs produced *via* bacterial metabolite synthesis exhibit antioxidant and anti-inflammatory properties. Researchers analyze these particles using XRD, TEM, and zeta potential measurements to determine structural and surface characteristics. These nanoparticles show promise for medical treatments due to their antioxidant and anti-inflammatory effects. Biologically synthesized Cu_2_Se materials kill bacteria after surface modifications and work against multiple bacterial strains.^20^ Comparative studies found biogenic CuO and Se monometallic nanoparticles required approximately 100 µg per mL (CuO) and 125 µg per mL (Se) concentrations to inhibit *S. aureus* and *E. coli* growth, though CuO eliminated bacteria faster (3–3.5 hours) than Se (4–5 hours).^21^ Furthermore, biogenic CuO nanoparticles from *Stenotrophomonas* extracts suppressed colon and gastric cancer cells but spared healthy cells, and PCR data showed changes in apoptosis genes P53, BAX, BCL2, and CCND1.^22^

Cu–Se NPs prevent plaque formation and inflammation in atherosclerosis by lowering cellular ROS levels,^23^ whereas Cu_2_Se nanoparticles improve wound healing through increased angiogenesis and fibroblast migration that repairs tissue.^24^ Furthermore, such nanoparticles within hydrogels treat dry eye disease by reducing oxidative damage and inflammation.^25^ Separately, topically applied myco-synthesized selenium nanoparticles decreased wound size, bacterial counts, and IL-6 plus TNF-α levels in mouse studies, indicating both antimicrobial and healing properties for biogenic selenium nanoparticles.^20^ Most Se–Cu NPs research has used chemical coprecipitation or fungal synthesis, with *Pantoea agglomerans* requiring chemical Cu^+^ reduction,^26^ and *Aspergillus niger* applications limited to agriculture.^27^ These studies tested either antimicrobial or antioxidant properties separately, leaving the combined therapeutic potential unexplored. No previous work has employed Red Sea marine *Bacillus* species for bimetallic synthesis or evaluated antioxidant, anti-inflammatory, antimicrobial, and antibiofilm activities together against ATCC-standard pathogen panels.

This work targets the green synthesis of selenium–copper bimetallic nanoparticles (Se–Cu BMNPs) using metabolic extracts from the marine bacterium *Bacillus licheniformis* LHG166 isolated from Red Sea coastal waters, where bacterial metabolites function as both reducing and capping agents to replace toxic chemical synthesis routes. The biosynthesized nanoparticles are characterized through UV-Vis, FT-IR, XRD, TEM, EDX, DLS, and zeta potential analyses. Biomedical assessment evaluated the antioxidant capacity using DPPH and ABTS radical scavenging assays, anti-inflammatory activity through COX-1 and COX-2 enzyme inhibition screening, and antimicrobial performance against ATCC- Gram-positive bacteria (*B. subtilis*, *S. aureus*, *E. faecalis*), Gram-negative bacteria (*E. coli*, *P. aeruginosa*, *S. typhi*), yeasts (*C. albicans*, *C. tropicalis*), and filamentous fungi (*F. oxysporum*, *A. niger*) *via* agar well diffusion followed by broth microdilution to determine MICs and MBCs/MFCs with ratio calculations. Biofilm disruption is quantified through microtitre plate assays using crystal violet staining and spectrophotometric measurement to assess inhibition patterns at sub-lethal concentrations (25%, 50%, 75% of MBC) across the tested microbial panel.

## Materials and methods

### Bacterial extract preparation and biogenic Se–Cu BMNPs synthesis

The marine bacterium *Bacillus licheniformis* LHG166 (GenBank accession number OR906129.1) was isolated from Red Sea shores and identified through 16S rRNA gene sequencing. Our previous work focused on extracting its exopolysaccharide (EPSR2), investigating its chemical composition, and evaluating its biomedical properties.^28^

For metabolite extraction, bacterial cultures were grown aerobically in marine broth at 37 °C for 72 hours under shaking conditions (150 rpm). Cell-free supernatant was obtained by centrifugation at 8000 rpm for 15 minutes. The supernatant was mixed with ethyl acetate at a 1 : 1 (v/v) ratio in a separating funnel and shaken vigorously for 10 minutes. After phase separation, the organic layer was collected, and the extraction was repeated twice. The combined ethyl acetate fractions were evaporated under reduced pressure using a rotary evaporator at 40 °C. The concentrated extract was reconstituted in minimal ethyl acetate and stored at 4 °C until use.^29^

For Se–Cu BMNPs synthesis, 200 mL of aqueous solution containing 10 mM Na_2_SeO_3_ and 0.1 M CuSO_4_·5H_2_O was maintained at 30 °C under magnetic stirring at 500 rpm. The bacterial extract (20 mL) was added dropwise at 1.0 mL every 5 minutes. Stirring continued for 24 hours at room temperature to complete the bioreduction process. The precipitated nanoparticles were recovered by centrifugation and washed three times each with ethanol and acetone to remove organic residues. The purified BMNPs were dried in an oven at 50 °C for 6 hours.^30^

### Se–Cu BMNPs chemical characterization

Se–Cu BMNPs were dried at 45 °C for 24 hours, then stored in a desiccator and mixed with KBr. A thin disc of the sample-KBr mixture was examined using a Nicolet 6700 FT-IR spectrometer (Thermo Fisher Scientific) from 400–4000 cm^−1^ to identify functional group absorption bands.^31^ XRD patterns of Se–Cu BMNPs were captured using a PANalytical X'Pert Pro MRD diffractometer with CuKα radiation (*λ* = 1.54 Å). Diffraction data were collected from 10° to 80° (2*θ*) at 40 kV and 30 mA, revealing the crystalline structure of the synthesized nanoparticles.^32^ TEM analysis using JEOL Ltd-1010 (Tokyo, Japan) examined the size distribution and morphology of the Se–Cu BMNPs. The powder was ultrasonicated in water, drop-cast onto carbon-coated TEM grids, air-dried, and viewed under the microscope.^33^ The elemental makeup of biosynthesized Se–Cu BMNPs was investigated through energy-dispersive X-ray spectroscopy using a JEOL JSM6360LA apparatus. DLS analysis using Malvern Nano-ZS (Malvern Ltd, UK) measured the sample size distribution in colloidal suspension. Nanoparticles were dispersed in MilliQ water to reduce signal interference. The Malvern Nano-ZS Zeta-sizer determined surface charge properties under the same conditions.^34^

### Antioxidant evaluation

#### DPPH assessment

A 0.1 mM DPPH solution in C_2_H_5_OH was prepared. 1 mL of this solution was mixed with 3 mL of Se–Cu BMNPs solutions at varying concentrations (1.9–1000 µg mL^−1^), prepared by diluting ethanol-soluble samples. The mixtures were shaken and incubated at room temperature for 30 minutes alongside ascorbic acid as a positive control. Absorbance was measured at 517 nm using a UV-VIS Milton Roy spectrophotometer.^35^DPPH scavenging % = [(ascorbic acid_absorbance_ − Se–Cu BMNPs_absorbance_)/ascorbic acid_absorbance_] × 100

#### ABTS˙^+^ scavenging test

ABTS radical cation was generated by mixing 7 mM ABTS with 2.45 mM K_2_S_2_O_8_, then stored in darkness. 0.07 mL of the sample were combined with 3 mL diluted ABTS˙^+^ solution. After 6-minute incubation, absorbance was measured at 734 nm using spectrophotometry with ascorbic acid as a positive control.^36^ABTS˙^+^ inhibition % = [(ascorbic acid_absorbance_ − Se–Cu BMNPs_absorbance_)/ascorbic acid_absorbance_] × 100

### COX enzyme inhibition assay

The assessment of the sample's capacity to inhibit COX-1 and COX-2 isoenzymes was conducted using COX-1 (catalog no. k548) and COX-2 (catalog no. k547) inhibitor screening assay kits (Biovision, USA).^37^ The sample was dissolved in DMSO and tested at concentrations ranging from 1000 to 0.5 µg mL^−1^ in a final volume of 1 mL. Celecoxib served as a positive control for both COX-1 and COX-2 inhibition assays.^38^COX-inhibition % = [(celecoxib − Se–Cu BMNPs)/celecoxib] × 100

### Antimicrobial evaluation of the biogenic Se–Cu BMNPs

The agar well diffusion technique was employed to test how effectively Se–Cu BMNPs inhibit bacterial growth across various bacterial species from the ATCC collection. Tests were performed on Gram-positive bacteria (*Bacillus subtilis* ATCC 6633, *Staphylococcus aureus* ATCC 6538, and *Enterococcus faecalis* ATCC 29212) alongside Gram-negative bacteria (*Escherichia coli* ATCC 8739, *Pseudomonas aeruginosa* ATCC 90274, and *Salmonella typhi* ATCC 6539) using Mueller-Hinton agar media (Oxoid, UK). Fungal susceptibility testing was also conducted on sabouraud dextrose agar (Difco, USA) with *Fusarium oxysporum* ATCC strain 46995 and *Aspergillus niger* ATCC 16888, while *Candida albicans* ATCC 10221 and *Candida tropicalis* 66029. Gentamicin acted as the bacterial control,^39^ and fluconazole as the fungal control during screening.^40^

The inoculum suspension was made using the standard broth dilution protocol and applied to agar plates within 15 minutes. Streaking occurred in three directions across the dried agar surface for uniform coverage. After 15 minutes of complete drying, a sterile cork borer (6 mm diameter) punched wells into the agar under aseptic conditions.^41^ Se–Cu BMNPs dissolved in DMSO at 10 µg mL^−1^ were dispensed at 100 µL per well.^42^ Incubation conditions varied according to microorganism type, bacterial strains were incubated at 37 °C for 24 hours, *Candida* species at 35 °C for 48 hours, while *Fusarium oxysporum* and *Aspergillus niger* required 48–72 hours at 28 °C. Inhibition zones were measured to the nearest millimetre at points showing substantial growth reduction. CLSI protocols determined MICs and MBCs.^43^

### Antibiofilm assessment of Se–Cu BMNPs

The impact of Se–Cu BMNPs on biofilm development was examined using 96-well polystyrene plates. Trypticase soy yeast broth (TSY, 300 µL) containing 10^6^ CFU mL^−1^ bacteria was treated with sub-inhibitory concentrations of Se–Cu BMNPs at 75%, 50%, and 25% of the MBC. After 48 hours of aerobic static incubation at 37 °C, biofilms formed on the plates were stained with crystal violet for 15 minutes. Excess stain was removed by washing with sterile dH_2_O. Stained cells were solubilized using 250 µL of 95% ethanol per well. After 15 minutes, absorbance readings at 570 nm were taken with a microplate reader to quantify biofilm formation.^44^Biofilm inhibition (%) = 1 − [(*A*(sample) − *A*(blank))/(*A*(control) − *A*(blank))] × 100*A*(sample), *A*(blank), and *A*(control) indicate absorbance at 570 nm for wells with media plus organism plus Se–Cu BMNPs (at 25%, 50%, or 75% MBC), media alone, and media plus organism, respectively.

### Statistical analysis

SPSS software (version 22, USA) was used for all statistical computations. The Shapiro–Wilk test evaluated data distribution, while Levene's test checked variance homogeneity. Group comparisons were conducted through One-Way ANOVA. Each experiment was run three times (*n* = 3), with data reported as mean ± SD. Values of *p* < 0.05 were considered statistically significant. Tukey's HSD test enabled pairwise comparisons among treatment groups following significant ANOVA results.

## Results and discussion

### Biogenic synthesis and chemical characterization

**Fig. 1:**
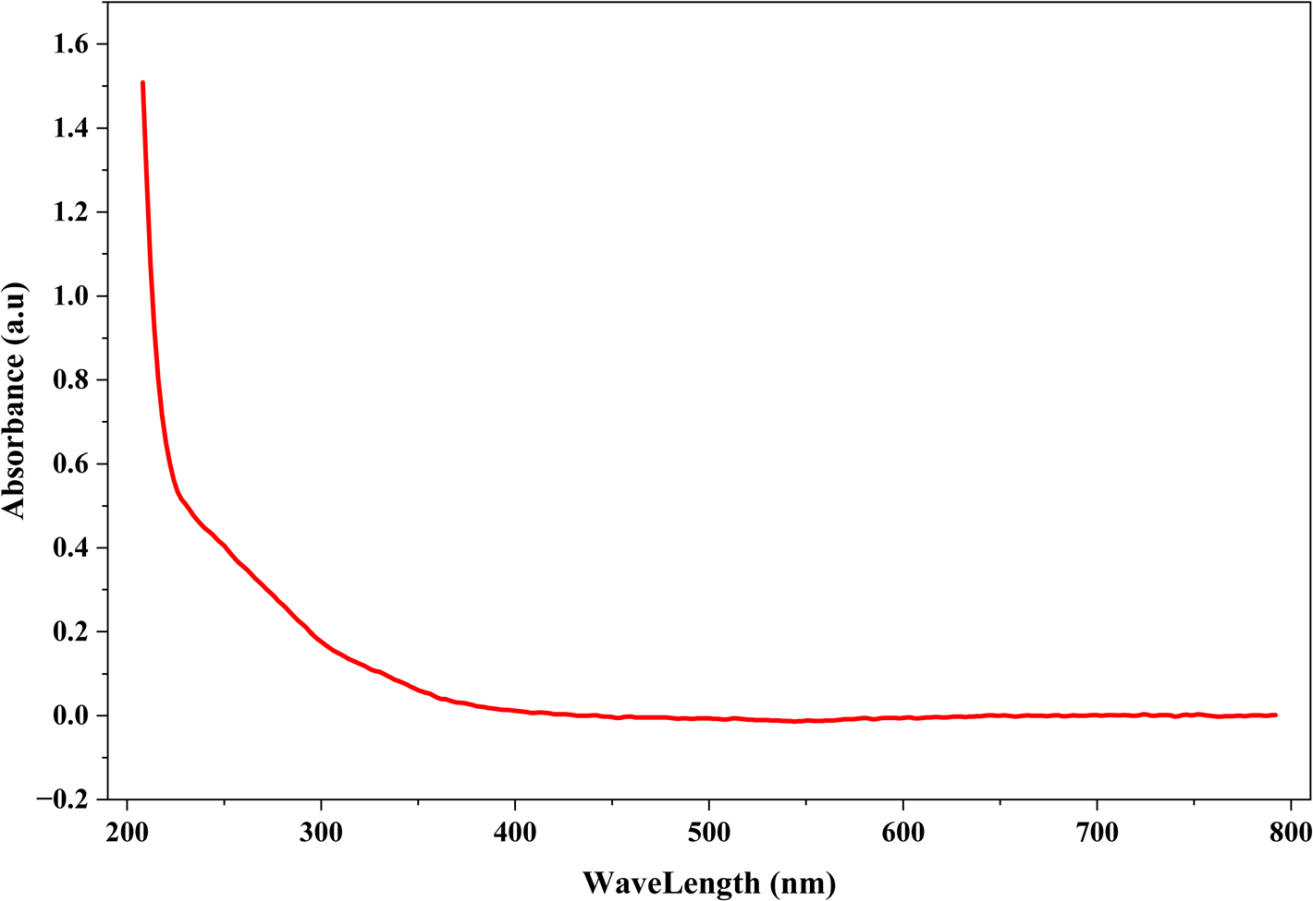
UV-Vis absorption spectrum of Se–Cu BMNPs.

The UV-Vis spectrum showed maximum absorption at 208 nm (1.508 absorbance units, a.u.) with exponential decay to 400 nm, dominated by selenium's interband electronic transitions (Fig. 1). No absorption was observed in the 570–600 nm region where metallic Cu^0^ surface plasmon resonance would occur. This absence indicates copper exists in oxidized states (Cu^+^ or Cu^2+^) rather than metallic form, as Cu_2_O and CuO do not exhibit characteristic plasmon bands in the visible range.

FT-IR spectroscopy was employed to identify the functional groups, comparative analysis of both spectra revealed significant peak shifts and intensity changes, indicating direct interaction between bioactive compounds and the nanoparticle surface. The bacterial extract spectrum exhibited characteristic protein signatures with prominent amide I absorption at 1621.96 cm^−1^ and polysaccharide features at 1016.56 cm^−1^, alongside aliphatic C–H stretching bands at 2925.32 and 2855.20 cm^−1^ (Fig. 1a).

**Fig. 2:**
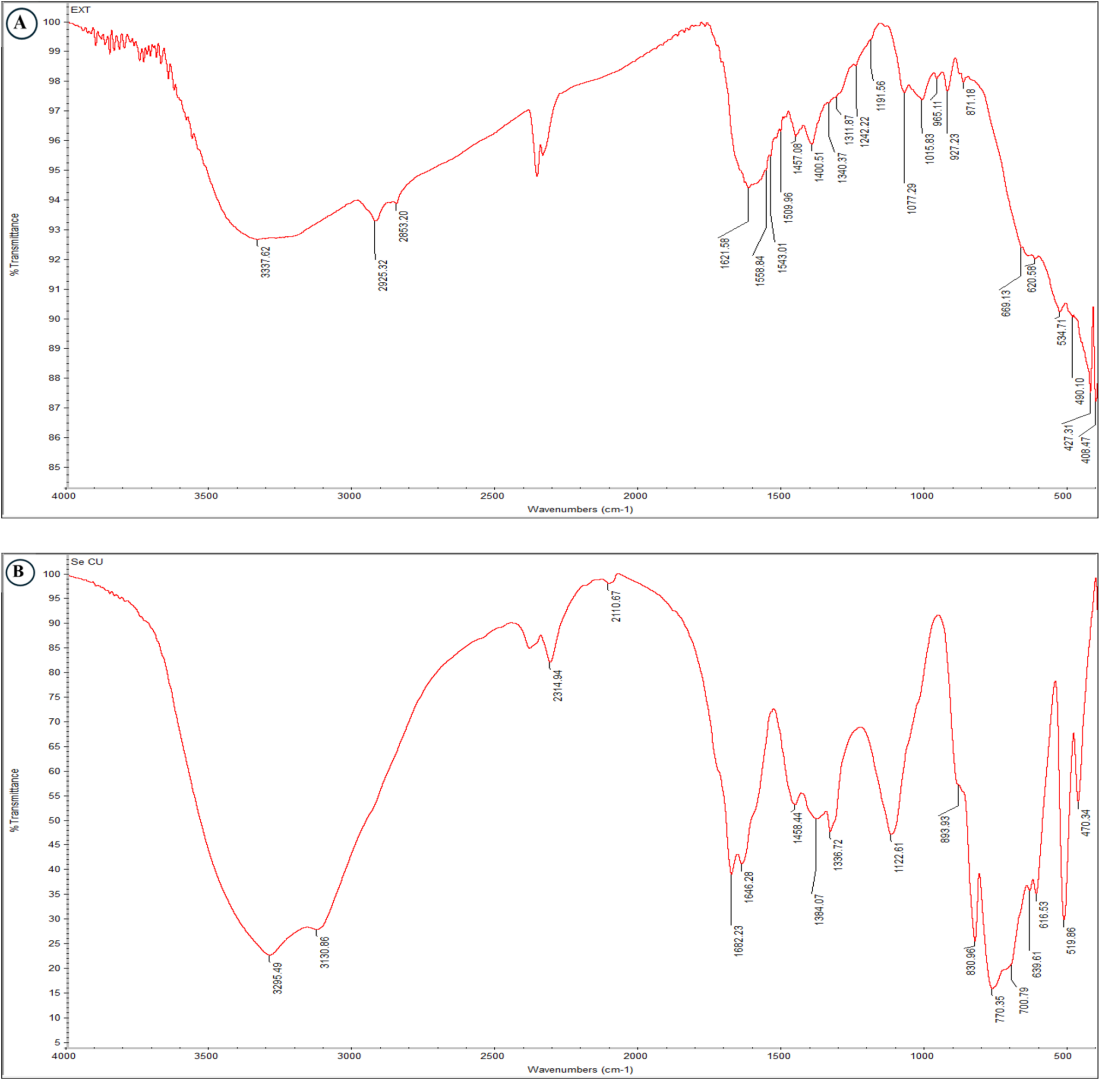
FT-IR spectra of bacterial extract (A) and Se–Cu BMNPs (B), with comparative peak assignments.

Following Se–Cu BMNPs synthesis, several peaks disappeared entirely (2925.32, 2855.20, 1556.34, 1457.09, 1342.25, 1191.56, 871.18 cm^−1^), demonstrating consumption or structural modification of these functional groups during metal reduction and nanoparticle formation. The amide I band shifted from 1621.96 to 1646.28 cm^−1^, confirming protein-mediated stabilization through carbonyl coordination with Cu^2+^ and Se^4+^ ions. Carboxylate stretching at 1400.51 cm^−1^ shifted to 1384.07 cm^−1^, while polysaccharide C–O vibrations moved from 1016.56 to 883.93 cm^−1^, both indicating metal–ligand complex formation on nanoparticle surfaces (Fig. 2b).

The hydroxyl/amine stretching region broadened and shifted from 3357.64 to 3295.49 cm^−1^, revealing extensive hydrogen bonding networks that enhance colloidal stability. Most significantly, a new absorption band emerged at 470.34 cm^−1^ in the BMNPs spectrum, corresponding to Cu–O and Se–O stretching vibrations and providing direct spectroscopic evidence for successful bimetallic nanoparticle synthesis with metal–oxygen bonding character (Fig. 2b and Table 1).

**Table 1:** FT-IR peak assignments and shifts comparing bacterial extract and Se–Cu BMNPs spectra

Bacterial extract (cm^−1^)	Se–Cu BMNPs (cm^−1^)	Functional group	Vibrational mode	Peak shift (cm^−1^)	Interpretation
3357.64	3295.49	O–H, N–H	Stretching	−62.15	Hydrogen bonding between hydroxyl/amine groups and nanoparticle surface
2925.32	—	C–H (aliphatic)	Asymmetric stretching	Disappeared	Loss of aliphatic chains suggests oxidation during synthesis
2855.20	—	C–H (aliphatic)	Symmetric stretching	Disappeared	Removal of lipid components
1621.96	1646.28	C=O (amide I)	Stretching	+24.32	Coordination of carbonyl oxygen with Cu^2+^/Se ions
1556.34	—	N–H (amide II)	Bending	Disappeared	Protein denaturation or metal–nitrogen coordination
1543.81	1456.04	C–H, CH_2_	Bending	−87.77	Structural changes in the organic capping layer
1457.09	—	C–H	Bending	Merged	Integration into the broader peak region
1400.51	1384.07	COO^−^ (carboxylate)	Symmetric stretching	−16.44	Metal–carboxylate complex formation
1350.96	1381.72	C–N, C–O	Stretching	+30.76	Altered electronic environment from metal coordination
1219.67	1122.18	C–O	Stretching	−97.49	Polysaccharide involvement in stabilization
1342.25	—	S=O	Stretching	Disappeared	Sulfur compounds participated in metal reduction
1191.56	—	P=O	Stretching	Disappeared	Phosphate groups involved in nucleation
1016.56	883.93	C–O–C	Stretching	−132.63	Glycosidic linkages interacting with the metal surface
927.23	830.98	C–H	Out-of-plane bending	−96.25	Conformational changes in aromatic residues
871.18	—	C–O–C	Stretching	Disappeared	Sugar ring deformation during capping
865.11	—	C–C	Stretching	Disappeared	Structural rearrangement of the organic matrix
1077.28	—	C–O, P–O	Stretching	Disappeared	Involvement in the chelation mechanism
668.19	700.79	C–S, C–Cl	Stretching	+32.60	Residual organic groups on nanoparticle periphery
620.52	616.53	Metal–O	Stretching	−3.99	Weak Cu–O or Se–O bonding
534.71	519.96	Metal–O	Bending	−14.75	Formation of metal–oxygen bonds
427.31	470.34	Cu–O, Se–O	Stretching	+43.03	Confirms Se–Cu BMNPs formation
409.87	—	Metal–ligand	Stretching	Merged	Metal–organic coordination bonds
—	3130.86	O–H	Stretching	New peak	Residual moisture or surface hydroxylation
—	2314.94	—	—	New peak	Possible CO_2_ absorption or artifact
—	2110.67	C≡C, C≡N	Stretching	New peak	Trace nitrile/alkyne groups from thermal treatment
—	1852.23	C=O (ester)	Stretching	New peak	Esterification during synthesis or drying

**Fig. 3:**
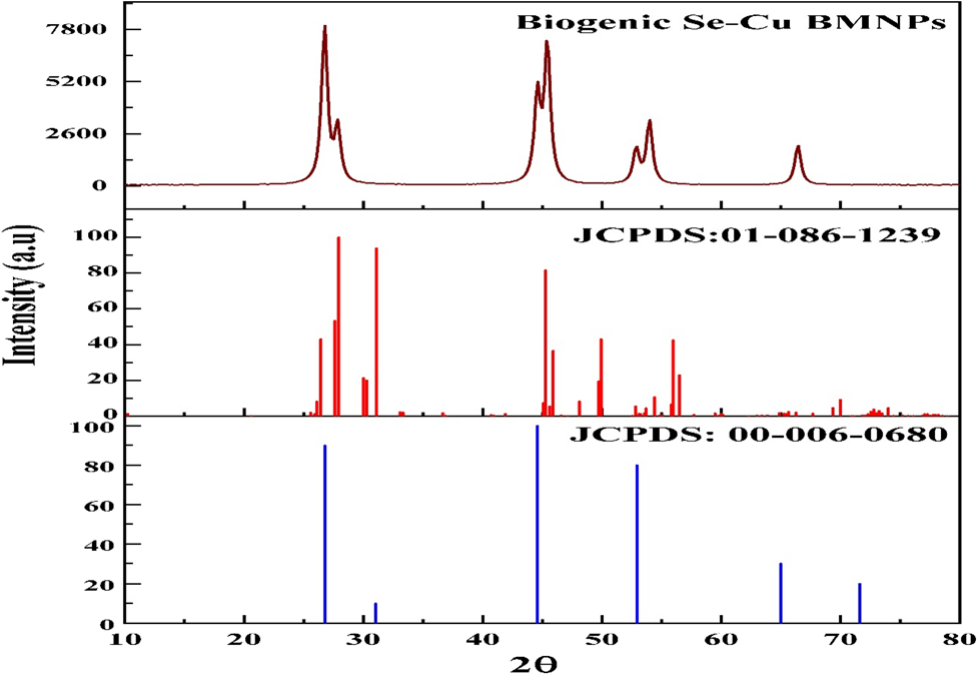
XRD pattern of Se–Cu BMNPs showing characteristic diffraction peaks at 2*θ* value.

XRD analysis confirmed the crystalline structure of the biosynthesized Se–Cu BMNPs. The diffractogram exhibited sharp, well-defined peaks at 2*θ* values of 26.7°, 27.7°, 44.5°, 45.3°, 52.9°, 53.9°, and 66.4° (Fig. 3). These reflections corresponded to the (111), (022), (220), (117), (311), (042), and (227) crystal planes, respectively. On matching these diffractions with the standard JCPDS cards, these reflections matched well with the orthorhombic (JCPDS No. 01-086-1239) and cubic (JCPDS No. 00-006-0680) phases of copper selenide (CuSe). Similar observations were previously reported for the hexagonal CuSe nanostructure.^45^

Crystallite size calculated using the Debye–Scherrer equation^46^ from the three dominant peaks yielded an average value of 27.2 nm, suggesting nanocrystalline dimensions of the prepared CuSe. The absence of any reflections corresponding to Cu, CuO, Cu_2_O, or Se confirmed the phase purity and successful formation of the CuSe bimetallic compound through biogenic synthesis.

**Fig. 4:**
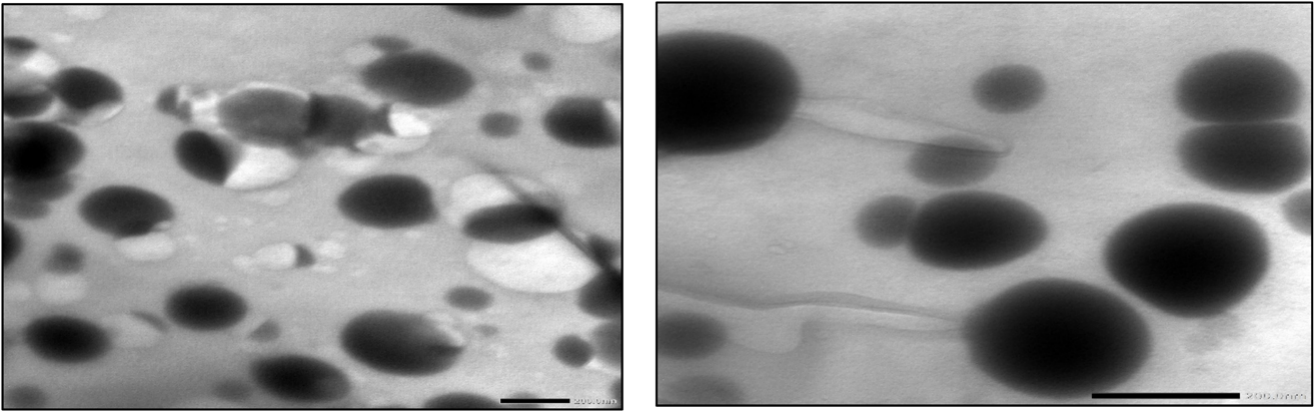
TEM images of spherical Se–Cu bimetallic nanoparticles with diameters ranging from 30–120 nm.

The biosynthesized Se–Cu BMNPs exhibited spherical to quasi-spherical morphologies with diameters ranging from 20–120 nm, demonstrating polydisperse size distribution throughout the sample (Fig. 4). Particles displayed electron-dense cores with uniform internal contrast, indicating homogeneous metal distribution. Most particles maintained smooth, rounded edges, while occasional irregular shapes appeared where smaller units had fused. Both compact and larger spherical particles are visible throughout the sample.

**Fig. 5:**
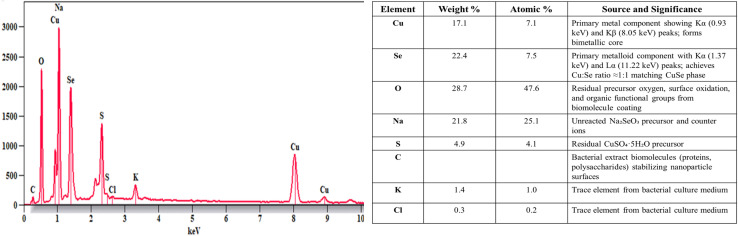
EDX spectrum of biogenic Se–Cu BMNPs.

EDX spectroscopy verified the elemental composition of the biosynthesized nanoparticles. Characteristic peaks appeared for copper (Kα at 0.93 keV, Kβ at 8.05 keV) and selenium (Kα at 1.37 keV, Lα at 11.22 keV), confirming both metals were incorporated during synthesis. Quantitative analysis showed copper at 17.1 wt% (7.1 at%) and selenium at 22.4 wt% (7.5 at%); the difference between weight and atomic percentages reflects the difference in atomic masses (Cu: 63.55 g mol^−1^*vs.* Se: 78.97 g mol^−1^), producing a Cu : Se atomic ratio near 1 : 1 that matches the CuSe phase from XRD measurements. Carbon signal (3.3 wt%) came from bacterial extract biomolecules on nanoparticle surfaces, matching FT-IR evidence of protein and polysaccharide stabilization. Sodium (21.8 wt%), oxygen (28.7 wt%), and sulfur (4.9 wt%) remained from unreacted Na_2_SeO_3_ and CuSO_4_·5H_2_O precursors with their counter ions (Fig. 5).

Oxygen content also reflects surface oxidation and contributions from organic functional groups in biomolecule layers. Potassium (1.4 wt%) and chlorine (0.3 wt%) traces originated from bacterial culture medium. The Cu : Se stoichiometric ratio validates CuSe bimetallic phase formation as the main nanoparticle composition.

**Fig. 6:**
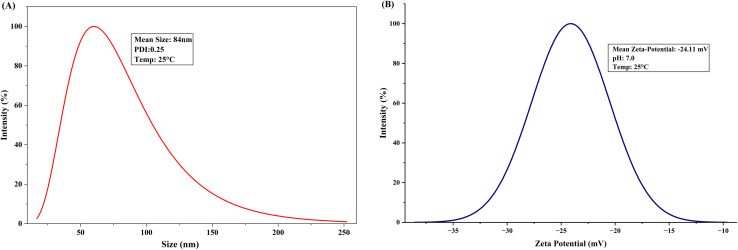
(A) Particle size distribution of Se–Cu BMNPs measured by DLS. (B) Zeta potential distribution displaying surface charge of Se–Cu BMNPs.

DLS analysis revealed a mean hydrodynamic diameter of 84 nm with a polydispersity index of 0.26, measured at 25 °C. The PDI value below 0.3 indicates reasonably uniform size distribution, though some heterogeneity remains in the nanoparticle population. The particle size distribution curve showed a broad peak spanning approximately 20–200 nm, with maximum intensity near 84 nm, confirming nanoscale dimensions (Fig. 6a).

Zeta potential measurements yielded a value of −24.11 mV in deionized water at pH 7.0, demonstrating moderate negative surface charge (Fig. 6b). This negative charge derives from deprotonated carboxyl (–COO^−^) and hydroxyl groups in bacterial exopolysaccharides and proteins coating the nanoparticle surface, as confirmed by FT-IR spectroscopy.

Marine bacteria make Se–Cu NPs by using their metabolic processes to reduce and stabilize these particles. The bacteria's proteins and metabolites act as reducing and capping agents that keep the nanoparticles stable.^47^ FTIR analysis detected proteinaceous compounds in the synthesized SeNPs, which confirms that bacterial proteins take part in nanoparticle formation.^34^ More researchers are turning to this biological method because it doesn't harm the environment and has potential medical applications. For example, *Kocuria flava* synthesizes copper NPs through biomineralization, as other marine microbes do with metals.^48^*B. licheniformis* changes toxic selenium oxyanions into stable biogenic selenium nanoparticles (BioSeNPs) that can be used alongside copper.^49^*Lactobacillus acidophilus* creates selenium and copper oxide nanoparticles, these nanoparticles stopped food spoilage microorganisms from growing.^50^

The amount of the NPs generated depends heavily on nutrient levels and how long the reaction runs. For instance, *B. amyloliquefaciens* synthesizes the most SeNPs when selenite oxyanion is at 2 mM, cell biomass reached 20 g L^−1^ (wet weight), and the mixture sat for 60 hours.^50^*P. agglomerans* synthesized cuprous selenide (Cu_2_Se) nanospheres through a biological–chemical reduction process, where Cu^+^ ions catalyze Se^2−^ ion formation.^51^*L. acidophilus* made SeNPs and CuONPs that turned the solution a different color as they formed, sized 75.52–153.22 nm with shapes from spherical to vaulted.^52^*E. coli* and *P. aeruginosa* produced SeNPs of 90–150 nm, most of them spherical.^53^*E. faecalis* created smaller copper nanoparticles at 20–90 nm that dispersed evenly and reached concentrations of about 6.52 × 10^10^ particles per ml.^54^ Similarly, CuO–Se BNPs made from *L. siceraria* leaf extract had a core–shell structure, spherical shape, and measured 50 nm in size.^55^ CuNPs made from *Klebsiella pneumoniae* strain NST2 had crystallite sizes between 22.44 nm and 44.26 nm, with carbonyl and amine functional groups keeping them stable.^56^ Another bimetallic NPs synthesized by *P. aeruginosa* from clinical specimens, measured 83–91 nm in diameter with spherical shape, had a −17.6 mV negative surface charge, and used proteins as capping agents, shown by UV-Vis, FTIR, zeta potential, and TEM.^57^

### Antioxidant evaluation of the biogenic Se–Cu BMNPs

The antioxidant activity of biogenic Se–Cu BMNPs was measured using the DPPH radical scavenging assay, with ascorbic acid serving as the reference standard. Both compounds scavenged free radicals more effectively as concentrations increased. At 1.9 µg mL^−1^, Se–Cu BMNPs scavenged 40.5% of radicals, while ascorbic acid reached 48.1%. This gap persisted at 31.2 µg mL^−1^, where the values were 70.5% and 72.2% respectively. Higher concentrations showed the nanoparticles catching up: at 125 µg mL^−1^, Se–Cu BMNPs achieved 82.8% *vs.* ascorbic acid's 85.3%, rising to 93.2% and 95.5% at 500 µg mL^−1^. At the maximum tested concentration of 1000 µg mL^−1^, Se–Cu BMNPs scavenged 96.1% of radicals compared to ascorbic acid's 98.0% (Table 2).

**Table 2:** DPPH and ABTS radical scavenging activity and IC_50_ values of Se–Cu BMNPs and ascorbic acid at concentrations ranging from 1.9 to 1000 µg mL^−1^

Conc. (µg mL^−1^)	Antioxidant scavenging activity			
	Se–Cu BMNPs DPPH scavenging % IC_50_ = 4.1 µg mL^−1^	Ascorbic acid DPPH scavenging % IC_50_ = 3.1 µg mL^−1^	Se–Cu BMNPs ABTS˙^+^ scavenging % IC_50_ = 10.73 µg mL^−1^	ABTS˙^+^ ascorbic acid scavenging % IC_50_ = 2.55 µg mL^−1^
1.9	40.5	42.5	30.6	48.1
3.9	48.4	51.6	40.2	53.8
7.8	56.8	59.1	46.5	58.5
15.6	62.6	65.5	55.4	63.8
31.2	70.5	72.2	60.9	69.9
62.5	76.8	79.8	68.4	77.1
125	82.8	85.3	75.4	81.1
250	88.8	92.2	84.3	87.5
500	93.2	95.5	88.5	94.3
1000	96.1	98	94.6	96.8

Statistical analysis revealed no significant difference (*p* > 0.05) between Se–Cu BMNPs and ascorbic acid at concentrations ≥500 µg mL^−1^ for DPPH scavenging, while lower concentrations showed significantly higher activity for ascorbic acid (*p* < 0.05).

The IC_50_ values reflected this pattern, with Se–Cu BMNPs at 4.1 µg mL^−1^ and ascorbic acid at 3.1 µg mL^−1^. The bimetallic nanoparticles, therefore, matched ascorbic acid's antioxidant strength quite closely, particularly at higher concentrations, indicating their viability as free radical scavengers.

Moving to the ABTS evaluation assay, the biogenic Se–Cu BMNPs scavenged ABTS^.+^ cation radicals at every concentration tested. At 1.9 µg mL^−1^, the nanoparticles reached 30.6% inhibition, while ascorbic acid achieved 48.1%. At 7.8 µg mL^−1^, the values were 46.5% and 58.5%, and at 31.2 µg mL^−1^, they reached 60.9% and 69.9%. At 125 µg mL^−1^, Se–Cu BMNPs achieved 75.4% *vs.* ascorbic acid's 81.1%, then 88.5% *versus* 94.3% at 500 µg mL^−1^, and 94.6% *versus* 96.8% at 1000 µg mL^−1^ (Table 2). All experiments were performed in triplicate (*n* = 3). One-Way ANOVA showed significant differences between concentrations (*p* < 0.001). The IC_50_ values of 4.1 ± 0.3 µg per mL (Se–Cu BMNPs) *vs.* 3.1 ± 0.2 µg per mL (ascorbic acid) for DPPH, and 10.73 ± 0.8 µg mL^−1^*vs.* 2.55 ± 0.4 µg mL^−1^ for ABTS, revealed no significant difference at 500 and 1000 µg per mL concentrations (*p* > 0.05). The IC_50_ values showed Se–Cu BMNPs at 10.73 µg mL^−1^ compared to ascorbic acid's 2.55 µg mL^−1^. The nanoparticles still reached over 88% scavenging at higher concentrations, matching closely with ascorbic acid's performance and confirming their effective antioxidant capacity in neutralizing ABTS radicals.

Se–Cu BMNPs show strong antioxidant effects against DPPH and ABTS radicals due to their distinct structural features and multiple working mechanisms. These nanoparticles scavenge radicals effectively through intramolecular charge transfer between Cu(i)/Cu(ii) and Se redox couples, which facilitates electron donation to free radicals and neutralizes them.^58^ The copper vacancies present in Cu_2−*x*_Se NPs also catalyze oxidation reactions, a crucial factor in their antioxidant performance.^59^ Additionally, copper(ii) complexes scavenge DPPH and ABTS radicals by donating electrons or hydrogen atoms, which stops the propagation of radical reactions.^60^ These complexes can function as either antioxidants or prooxidants based on their concentration and environmental conditions, altering their biological activity after metabolism.^61^

Se–Cu BMNPs/NPs do more than just scavenge radicals directly, they also boost cellular antioxidant defenses by changing how oxidative stress genes are expressed and turning on specific signaling pathways.^62^ The nanoparticles increase production of antioxidative proteins like Nrf2, HO-1, and SOD2 while reducing NOX4 levels, which reinforces endogenous antioxidant mechanisms.^63^ They also improve the function of antioxidant enzymes such as glutathione peroxidase (GSH-Px) and superoxide dismutase, both of which help maintain redox balance by eliminating harmful reactive oxygen species (ROS) and affecting redox signaling routes.^64^

### Anti-inflammatory evaluation of the biogenic Se–Cu BMNPs

The biogenic Se–Cu BMNPs showed Cox-1 inhibition that increased with concentration. At 0.5 µg mL^−1^, the nanoparticles gave 19.5% inhibition against celecoxib's 21%, and at 1 µg mL^−1^ reached 28.5% *versus* 29%. Inhibition climbed to 37.2% at 2 µg mL^−1^, 43.8% at 3.9 µg mL^−1^, and 52% at 7.8 µg mL^−1^. At 15.6 µg mL^−1^, the Se–Cu BMNPs hit 60.3% inhibition, then 67.8% at 31.25 µg mL^−1^ and 76.7% at 62.5 µg mL^−1^. Higher concentrations gave 80.4% inhibition at 125 µg mL^−1^, 86.9% at 250 µg mL^−1^, 91.5% at 500 µg mL^−1^, and 97.3% at 1000 µg mL^−1^. Celecoxib values at these same concentrations were 21%, 29%, 36.8%, 45.3%, 55.5%, 64%, 71.3%, 79%, 83.9%, 88.1%, 92.4%, and 98.2%. The IC_50_ for Se–Cu BMNPs came to 7.05 ± 0.2 µg mL^−1^, while celecoxib's IC_50_ was 5.93 ± 0.5 µg mL^−1^ (Table 3). The nanoparticles matched celecoxib's anti-inflammatory action closely, with both reaching above 90% inhibition at the upper concentration range.

**Table 3:** Cox-1 and Cox-2 inhibition percentages of Se–Cu BMNPs and celecoxib (0.5–1000 µg mL^−1^) with calculated IC_50_ values

Conc. (µg mL^−1^)	Cox-inhibition assessment			
	Se–Cu BMNPs Cox-1 inhibition % IC_50_ = 7.05 ± 0.2 µg mL^−1^	Celecoxib Cox-1 inhibition % IC_50_ = 5.93 ± 0.5 µg mL^−1^	Se–Cu BMNPs Cox-2 inhibition % IC_50_ = 12.11 ± 0.9 µg mL^−1^	Celecoxib Cox-2 inhibition % IC_50_ = 4.51 ± 0.3 µg mL^−1^
0.5	19.5	21	15.9	23.5
1	28.5	29	23.1	32.9
2	37.2	36.8	30.3	40.5
3.9	43.8	45.3	39.5	48.2
7.8	52	55.5	47.4	56
15.6	60.3	64	52	65.2
31.25	67.8	71.3	59.2	75.7
62.5	76.7	79	67.4	81.9
125	80.4	83.9	76.5	85.7
250	86.9	88.1	81.5	89.3
500	91.5	92.4	88.3	94
1000	97.3	98.2	95.3	99

The biogenic Se–Cu BMNPs showed Cox-2 inhibition that increased with concentration. At 0.5 µg mL^−1^, the NPs gave 15.9% inhibition, while celecoxib showed 23.5%, and at 1 µg mL^−1^, reached 23.1% *vs.* 32.9%. Inhibition climbed to 30.3% at 2 µg mL^−1^ against celecoxib's 40.5%, then 39.5% at 3.9 µg mL^−1^*vs.* 48.2%, and 47.4% at 7.8 µg mL^−1^*vs.* 56%. At 15.6 µg mL^−1^, the Se–Cu BMNPs hit 52% with celecoxib at 65.2%, then 59.2% at 31.25 µg mL^−1^*vs.* 75.7%, and 67.4% at 62.5 µg mL^−1^*vs.* 81.9%. Higher concentrations gave 76.5% at 125 µg mL^−1^ with celecoxib at 85.7%, 81.5% at 250 µg mL^−1^*vs.* 89.3%, 88.3% at 500 µg mL^−1^*vs.* 94%, and 95.3% at 1000 µg mL^−1^*vs.* 99% (Table 3). The IC_50_ for Se–Cu BMNPs came to 12.11 ± 0.9 µg mL^−1^ while celecoxib's IC_50_ was 4.51 ± 0.3 µg mL^−1^. One-Way ANOVA revealed concentration-dependent inhibition for both enzymes (*p* < 0.001). Tukey's HSD *post-hoc* analysis showed Se–Cu BMNPs matched celecoxib performance at ≥250 µg mL^−1^ for COX-1 (*p* > 0.05) and ≥500 µg mL^−1^ for COX-2 (*p* > 0.05).

The NPs produced potent Cox-2 inhibition, surpassing 95% at the highest dose. Se–Cu interacts with COX-1 and COX-2 enzymes and blocks their function differently. COX-1 operates constantly, but COX-2 appears during inflammation, both produce prostaglandins. Se–Cu changes how COX-2 binds substrates and reshapes its active site, altering its catalytic function. Copper ions irreversibly inhibit COX-2 through stoichiometric binding that stops the enzyme from working. Se–Cu biogenic nanoparticles also fight inflammation by trapping reactive oxygen species (ROS), molecules that drive chronic inflammation, reducing oxidative damage in tissues.

### Antimicrobial and antibiofilm assessment of Se–Cu BMNPs

**Fig. 7:**
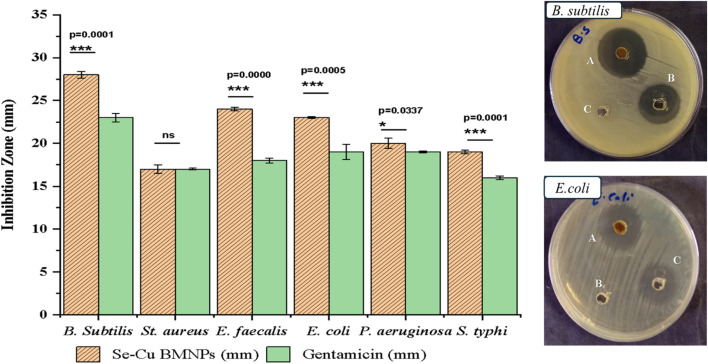
Comparative inhibition zones of Se–Cu BMNPs and gentamicin against bacterial pathogens.

**Fig. 8:**
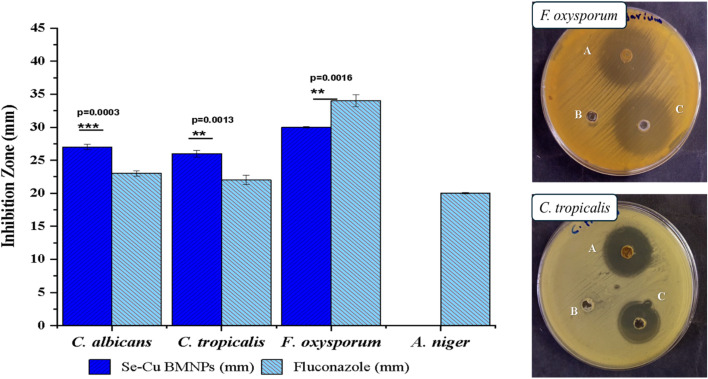
Comparative inhibition zones of Se–Cu BMNPs and fluconazole against filamentous molds and *Candida* species.

**Fig. 9:**
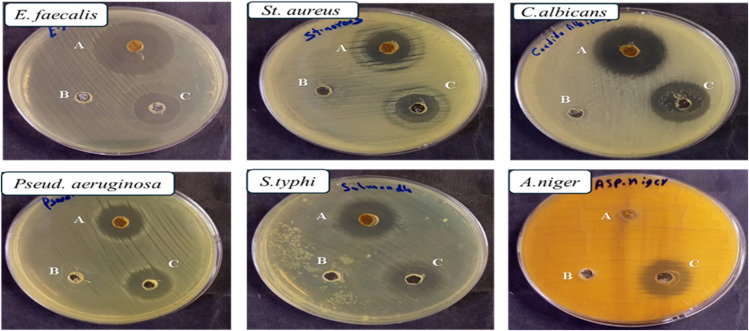
Inhibition zones represented in mm of Se–Cu BMNPs against bacteria and fungi. (A) Se–Cu BMNPs (B) blank (C) Gentamicin for bacteria/Fluconazole for fungi and *Candida* sp.

The G +ve organisms exhibited varying degrees of susceptibility to Se–Cu BMNPs, with *Bacillus subtilis* emerging as the most responsive strain across all measured parameters. This bacterium produced an inhibition zone of 28 ± 0.4 mm, which surpassed the gentamicin control by approximately 22%, while simultaneously requiring the lowest antimicrobial concentration at 15.62 µg mL^−1^ for both MIC and MBC values. The identical MIC and MBC readings translate to a ratio of 1.0, indicating immediate bactericidal action without any intermediate inhibitory phase. According to established microbiological criteria, MBC/MIC ratios ≤4 indicate bactericidal activity, while ratios >4 suggest bacteriostatic effects.^65^ All antimicrobial assays were performed in triplicate (*n* = 3). One-Way ANOVA confirmed significant susceptibility differences among organisms (*p* < 0.001). Se–Cu BMNPs produced larger zones than gentamicin against *B. subtilis* (28 ± 0.4 mm *vs.* 23 ± 0.2 mm, *p* = 0.0001), *E. faecalis* (24 ± 0.2 mm *vs.* 18 ± 0.3 mm, *p* < 0.001), and *E. coli* (23 ± 0.1 mm *vs.* 19 ± 0.9 mm, *p* < 0.05). *P. aeruginosa* showed 20 ± 0.5 mm for Se–Cu BMNPs *vs.* 19 ± 0.4 mm for gentamicin (*p* = 0.0337), while *S. typhi* demonstrated 19 ± 0.3 mm *vs.* 16 ± 0.2 mm (*p* = 0.0001). In contrast, *St. aureus* showed no significant difference (17 ± 0.6 mm *vs.* 17 ± 0.5 mm, *p* > 0.05). *Enterococcus faecalis* displayed considerable vulnerability as well, generating a 24 ± 0.2 mm zone compared to gentamicin's 18 ± 0.3 mm, a difference of 33%. However, this strain required a fourfold increase in concentration (31.25 µg mL^−1^) to inhibit growth, and another doubling to 62.5 µg mL^−1^ for complete eradication, yielding an MBC/MIC ratio of 2.0. *Staphylococcus aureus* presented the greatest challenge among G +ve bacteria, matching gentamicin's performance at 17 ± 0.5 mm but demanding substantially higher concentrations for both inhibitory and lethal effects. The MIC reached 125 µg mL^−1^, eight times higher than *B. subtilis*, while the MBC climbed to 250 µg mL^−1^, representing a sixteen-fold increase over the most susceptible organism. Despite these elevated concentration requirements, the 2.0 ratio confirms that Se–Cu BMNPs maintain bactericidal rather than merely bacteriostatic properties against this pathogen (Table 4 and Fig. 7–9).

**Table 4:** Antibacterial activity of Se–Cu BMNPs against pathogenic bacteria is represented as MIC and MBC^a^^,^^b^^,^^c^

ATCC pathogenic bacteria	MIC (µg ml^−1^)	MBC (µg ml^−1^)	MBC/MIC ratio
*Bacillus Subtilis* (ATCC 6633)	15.62	15.62	1
*Staphylococcus aureus* (ATCC 6538)	125	250	2
*Enterococcus faecalis* (ATCC 29212)	31.25	62.5	2
*Escherichia coli* (ATCC 8739)	31.25	62.5	2
*Pseud. aeruginosa* (ATCC90274)	31.25	62.5	2
*Salmonella typhi* (ATCC 6539)	62.5	125	2

a MIC: minimum inhibitory concentration.

b MBC: minimum bactericidal concentration.

c MBC/MIC ratio ≤4 indicates bactericidal activity.

On the other hand, G −ve bacteria demonstrated moderate to good sensitivity patterns, though generally requiring higher concentrations than their G +ve counterparts, with the notable exception of *B. subtilis*. *Escherichia coli* responded favorably with a 23 ± 0.1 mm inhibition zone, outperforming gentamicin's 19 ± 0.9 mm by 21%, and required 31.25 µg mL^−1^ for growth suppression. The bactericidal concentration doubled to 62.5 µg mL^−1^, producing an MBC/MIC ratio of 2.0 that confirms lethal activity at reasonably low concentrations. *Pseudomonas aeruginosa*, often problematic due to its intrinsic resistance mechanisms, showed only marginal superiority over the control with zones measuring 20 ± 0.6 mm *vs.* 19 ± 0.1 mm.

Nevertheless, the MIC matched *E. coli* at 31.25 µg mL^−1^, and the MBC similarly reached 62.5 µg mL^−1^, maintaining the 2.0 ratio characteristic of bactericidal compounds. *Salmonella typhi* produced an 18.8% improvement over gentamicin with a 19 ± 0.2 mm zone, yet this strain proved more resistant in concentration-dependent assays, necessitating 62.5 µg mL^−1^ for inhibition and 125 µg mL^−1^ for complete killing. The resulting 2.0 ratio still falls within bactericidal parameters.

Fungal pathogens exhibited a bimodal response pattern, with *Candida* species showing exceptional sensitivity while filamentous fungi displayed either resistance or paradoxical behavior. *Candida albicans* and *Candida tropicalis* both generated impressive inhibition zones of 27 ± 0.4 mm and 26 ± 0.5 mm, respectively, exceeding their fluconazole controls and matching the robust activity observed against *B. subtilis*. Remarkably, both yeast species required only 15.62 µg mL^−1^ for growth inhibition, positioning them among the most susceptible organisms in the entire panel. The fungicidal concentration increased to 31.25 µg mL^−1^ for both *Candida* strains, producing an MBC/MIC ratio of 2.0 that demonstrated decisive killing rather than mere growth suppression. *Fusarium oxysporum* presented a more complex picture, achieving a 30 ± 0.1 mm inhibition zone with Se–Cu BMNPs but generating an even larger 34 ± 0.9 mm zone with gentamicin, the only instance where the control outperformed the test material. This filamentous fungus required 31.25 µg mL^−1^ for inhibition but needed a substantial jump to 125 µg mL^−1^ for complete eradication, resulting in an MBC/MIC ratio of 4.0. While this ratio remains within the accepted threshold for fungicidal classification (≤4), it suggests a wider gap between growth suppression and organism death compared to other tested species. *Aspergillus niger* proved entirely resistant to Se–Cu BMNPs, showing no measurable inhibition zone while producing a 20 ± 0.1 mm zone against fluconazole, indicating that this particular mold possesses inherent mechanisms that neutralize or exclude the nanoparticle formulation. This resistance likely stems from *A. niger*'s melanin-rich cell wall structure, where melanin functions as a protective barrier that scavenges ROS and prevents NPs penetration.^66^ Melanin granules cross-linked with polysaccharides in the cell wall create a dense network that can intercept copper and selenium ions before they reach critical cellular targets.^67^ Furthermore, *A. niger* may utilize melanin as a sacrificial redox-active compound that neutralizes metal-induced oxidative stress, a mechanism documented for other antimicrobial agents (Table 5).^68^

**Table 5:** Antifungal assessment of Se–Cu BMNPs against filamentous molds and *Candida* represented as MIC MFC, and MFC/MIC ratio^a^^,^^b^^,^^c^

Tested filamentous molds &*Candida* sp.	Se–Cu BMNPs (mm)	Fluconazole control (mm)	MIC (µg ml^−1^)	MFC (µg ml^−1^)	MFC/MIC ratio
*Candida albicans* (ATCC 10221)	27 ± 0.4	23 ± 0.4	15.62	31.25	2
*Candida tropicalis* (ATCC 7349)	26 ± 0.5	22 ± 0.7	15.62	31.25	2
*Fusarium oxysporum* ATCC strain (46 995)	30 ± 0.1	34 ± 0.9	31.25	125	4
*Aspergillus niger* (ATCC 16888)	NA	20 ± 0.1	—	—	—

a MIC: minimum inhibitory concentration.

b MFC: minimum fungicidal concentration.

c MFC/MIC ratio ≤4 indicates fungicidal activity.

**Fig. 10:**
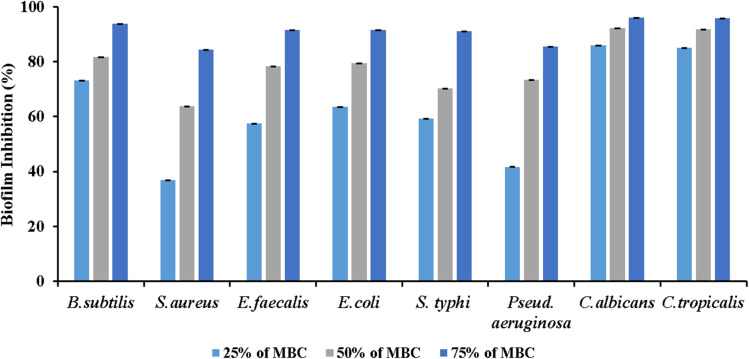
Antibiofilm activity of Se–Cu BMNPs against eight ATCC bacterial species and two *Candida* species at 25%, 50%, and 75% MBC.

Moving to antibiofilm evaluation of the biogenic Se–Cu BMNPs, the microtitre plate assay revealed concentration-dependent inhibitory effects of Se–Cu BMNPs across all tested microorganisms, though their efficacy varied considerably between species. Beginning with the 25% MBC concentration, the results demonstrated moderate activity, with *C. albicans* showing 85.95% inhibition, the highest among all organisms at this dilution. *B. subtilis* organism achieved 73.31% inhibition, while *E. faecalis* recorded 57.45%, and *Staphylococcus aureus* displayed only 36.81% effectiveness. The Gram-negative bacteria presented intermediate values, with *Pseudomonas aeruginosa* reaching 41.71%, *S. typhi* 59.37%, and *E. coli* 63.47%. At 50% MBC, the inhibitory capacity intensified across the board, with both *Candida* species maintaining superior performance, *C. albicans* at 92.22% and *C. tropicalis* at 91.73%. The bacterial strains showed substantial improvement, particularly *B. subtilis* (81.79%), *E. coli* (79.38%), and *E. faecalis* (78.25%), whereas *S. aureus* remained comparatively resistant at 63.86%. *Pseudomonas* and *Salmonella* recorded 73.43% and 70.29% respectively, indicating solid but not exceptional responses (Fig. 10) (Table 6).

Concentration-dependent antibiofilm efficacy of Se–Cu BMNPs against selected bacterial and fungal pathogens

**Table:** 

Se–Cu BMNPs – MBC% of *B. subtilis*	Anti-biofilm %	
Blank (media only)		
Media + organism	—	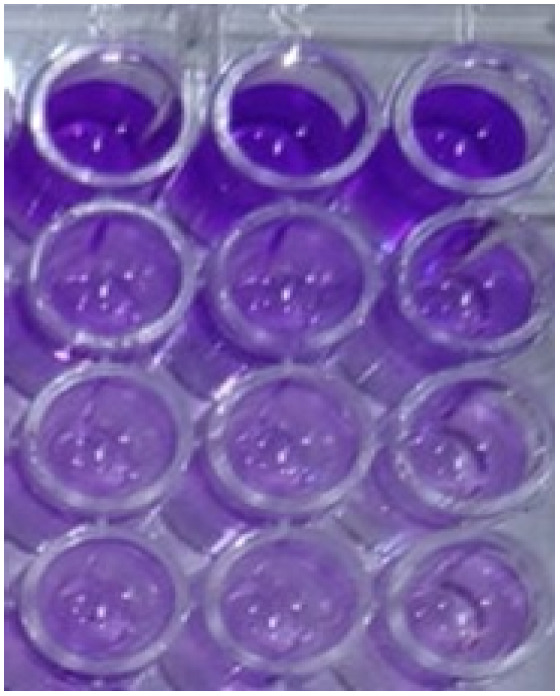
25% of MBC	73.31	
50% of MBC	81.79	
75% of MBC	93.76

**Table:** 

Se–Cu BMNPs – MBC% of *E. coli*	Anti-biofilm %	
Blank (media only)		
Media + organism	—	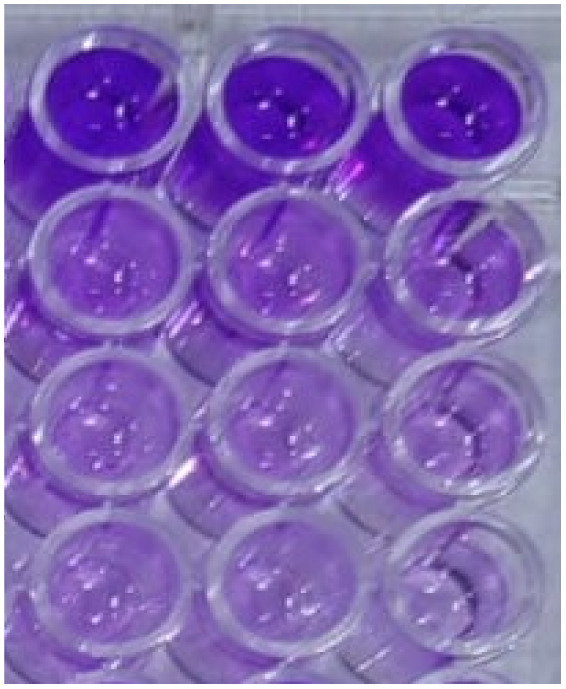
25% of MBC	63.47	
50% of MBC	79.38	
75% of MBC	91.59

**Table:** 

Se–Cu BMNPs – MBC% of *C. albicans*	Anti-biofilm %	
Blank (media only)		
Media + organism	—	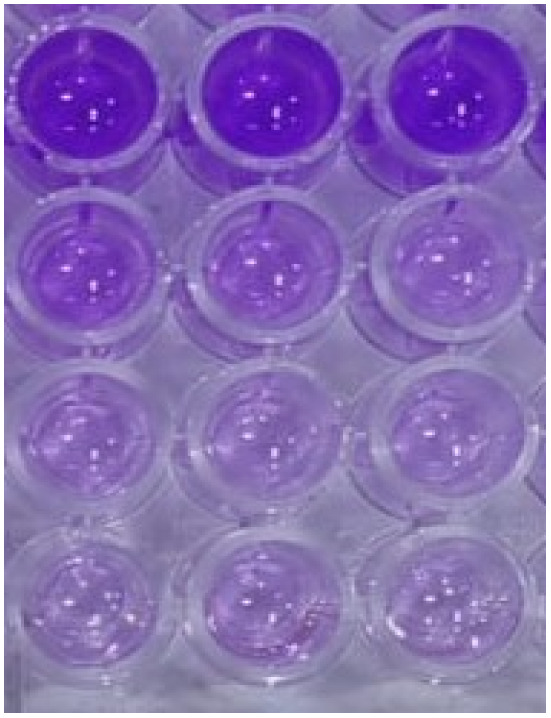
25% of MBC	96.09	
50% of MBC	92.22	
75% of MBC	91.59	

The 75% MBC concentration produced the most considerable results, with nearly all organisms demonstrating robust biofilm inhibition exceeding 90%. *C. albicans* topped the list at 96.09%, followed closely by *C. tropicalis* (95.84%), *B. subtilis* (93.76%), *E. coli* (91.59%), *E. faecalis* (91.60%), and *Salmonella typhi* (91.17%). Biofilm assays were performed in triplicate (*n* = 3). One-Way ANOVA demonstrated concentration-dependent effects across all organisms (*p* < 0.001). At 75% MBC, *C. albicans* and *C. tropicalis* showed higher inhibition (96.09 ± 1.2% and 95.84 ± 0.9%) than *S. aureus* (84.33 ± 2.1%, *p* < 0.01), reflecting distinct biofilm architectures.

Even the more resistant strains showed marked improvement, with *Pseudomonas* achieving 85.42% and *S. aureus* reaching 84.33% inhibition. *S. aureus* consistently proved most resistant among tested organisms, needing higher concentrations for substantial biofilm disruption, which reflects this pathogen's capacity to form resilient biofilm matrices. This resistance stems from multiple factors, *S. aureus* produces polysaccharide intercellular adhesin (PIA), a cationic exopolysaccharide that shields cells from antimicrobial agents,^69^ and incorporates extracellular DNA and cytoplasmic proteins into a dense matrix that physically blocks nanoparticle penetration.^70^ The bacterium also employs the Agr quorum-sensing system to regulate biofilm gene expression dynamically in response to cell density and environmental stress.^71^ These combined mechanisms create a robust defensive architecture that demands higher Se–Cu BMNPs concentrations to achieve comparable biofilm disruption.

The fungal species displayed exceptional susceptibility to Se–Cu BMNPs at all concentrations, pointing to particularly potent anti-fungal biofilm properties of these nanoparticles. *S. aureus* consistently proved most resistant among tested organisms, needing higher concentrations for substantial biofilm disruption, which reflects this pathogen's capacity to form resilient biofilm matrices.

Following our current findings, Cu–Se NPs synthesized by *Aspergillus niger* inhibited *Ralstonia solanacearum* at 12.5 µg mL^−1^, showing promise for antibacterial activity as well as for crop protection and fertilization.^72^ Se–Cu nanocomposites suppressed G +ve bacteria at 6.25–12.5 µg mL^−1^ and G −ve bacteria at 25–50 µg mL^−1^.^73^ In another study, selenium and copper oxide nanoparticles needed 125 µg mL^−1^ and 100 µg mL^−1^ to inhibit *Staphylococcus aureus* and *Escherichia coli*, with CuO proving more effective.^21^ Another biogenic bimetallic synthesized by watermelon rind extract killed fungal pathogens like *Candida albicans* and *Pestalotia* sp. at concentrations between 12.5 and 50 µg mL^−1^.^74^ In another study, a similar bimetallic generated by *Trichoderma harzianum* suppressed rice and wheat fungal pathogens as *Fusarium graminearum* and *Pyricularia oryzae*, at just 0.034 nM.^75^

It's worth mentioning that the physicochemical characteristics of biogenic Se–Cu bimetallic nanoparticles directly influence their antimicrobial activity. Se–Cu NPs form as crystallized nanospheres roughly 98.6 nm in diameter, a size that facilitates contact with microbial membranes.^73^ Their crystalline structure ensures stability and enables metal ion release, which drives antimicrobial action.^76^ Surface charge contributes too, where positively charged particles bind more readily to negatively charged bacterial walls, boosting their killing capacity.^77^ The bimetallic composition enables dual-target attack: copper disrupts membrane integrity and denatures nucleic acids through direct metal–protein interactions,^78^ while selenium simultaneously compromises thiol-dependent enzymes and interferes with sulfur metabolism pathways.^79^ This complementary mechanism prevents adaptive resistance that bacteria develop against single-metal systems.^80^ Against fungal pathogens, our NPs matched the lowest reported values, with *Candida* species requiring only 15.62 µg mL^−1^ compared to 12.5–50 µg mL^−1^ for watermelon rind-synthesized bimetallic NPs^81^ and 12.5 µg mL^−1^ for *Aspergillus*-derived Cu–Se NPs.^82^ The rapid bactericidal action (MBC/MIC ratios of 1.0–2.0) surpasses Ag–Cu bimetallic systems^83^ that typically show ratios of 2.0–4.0, indicating faster cell death kinetics.

Se–Cu BMNPs exert antimicrobial effects through multiple concurrent mechanisms. The particles adhere to microbial cell surfaces and compromise membrane and wall integrity, resulting in cytoplasmic leakage.^84^ Beyond structural damage, these nanoparticles stimulate intracellular ROS accumulation, which oxidizes lipids, proteins, and nucleic acids. Moreover, released copper ions interact with phosphorus- and sulfur-containing biomolecules, disrupting metabolic pathways and ATP generation.^85^ The nanoparticles bind to protein thiol groups and can replace sulfur atoms in cysteine and methionine residues, causing protein structural alterations.^86^ Additionally, genotoxic effects include DNA strand breaks and electron transport chain inhibition, which compromise cellular respiration.^87^

The dual-metal design delivers superior antimicrobial performance compared to single-metal particles. Copper denatures nucleic acids and proteins; selenium attacks selenoproteins and sulfur-based molecules, expanding the range of cellular targets.^88^ Beyond killing cells directly, Se–Cu nanoparticles diminish bacterial virulence by blocking production of pyocyanin, proteases, and pyoverdine, and by disrupting quorum-sensing networks.^89^ This prevents biofilm formation, a critical advantage against multidrug-resistant strains that hide within biofilm matrices.^90^ The metal synergy allows antimicrobial activity at lower doses than CuO or Se NPs require individually.^91^ Other bimetallic systems like Ag–Cu show similar improvements *via* combined ion release and ROS generation, though with reduced harm to mammalian cells.^92^

Future investigations should validate therapeutic efficacy through *in vivo* animal models, particularly for wound healing and systemic infections. Comprehensive toxicity profiling across acute and chronic exposures will establish safe dosage ranges, while pharmacokinetic studies must clarify biodistribution and elimination pathways. Scale-up optimization of bacterial synthesis needs attention to ensure reproducible nanoparticle characteristics for clinical applications. Formulation development into hydrogels or injectable preparations could enable topical and systemic delivery. Exploring synergistic effects with conventional antibiotics may enhance activity against these multidrug-resistant pathogens.

## Conclusion

This study demonstrates the successful one-step biogenic synthesis of crystalline CuSe bimetallic NPs with considerable biofilm disruption capacity (>90% inhibition at sub-lethal concentrations), addressing a critical therapeutic gap in treating resistant infections that conventional antibiotics fail to penetrate. Marine *Bacillus licheniformis* LHG166 extract successfully reduced metal precursors while simultaneously functionalizing NPs surfaces through protein and polysaccharide coatings. The biosynthesized BMNPs exhibited multifunctional therapeutic properties across antioxidant, anti-inflammatory, and antimicrobial domains. Free radical scavenging approached standard antioxidant performance, cyclooxygenase inhibition occurred at clinically relevant concentrations, and broad-spectrum antimicrobial activity emerged against bacterial and fungal pathogens. Strong biofilm disruption at sub-lethal doses addressed a critical challenge in treating resistant infections. The dual-metal composition enhanced these activities beyond single-metal systems by operating through complementary mechanisms, including membrane disruption, oxidative stress generation, and metabolic interference. Bacterial synthesis eliminates toxic reagents while creating inherently biocompatible materials with built-in surface functionalization, distinguishing this approach from conventional chemical methods.

Based on the current results, wound healing applications and biofilm-associated infection treatment represent the most promising candidates for near-term *in vivo* validation. The nanoparticles demonstrated robust activity against common wound pathogens (*S. aureus*, *E. coli*, *P. aeruginosa*), combined with potent antioxidant and anti-inflammatory properties that could accelerate tissue repair. Topical wound applications in diabetic ulcer models or burn infection models would provide direct evidence of therapeutic efficacy while minimizing systemic exposure concerns.

Several gaps require attention before clinical application. Acute and chronic toxicity studies must establish safe dosage ranges and identify organ-specific effects. Pharmacokinetic data, including biodistribution, clearance pathways, and tissue accumulation, remain absent. Scaling bacterial synthesis from 200 mL laboratory batches to production volumes while maintaining particle consistency presents technical hurdles. Testing synergistic effects with conventional antibiotics may reveal opportunities to reduce doses or expand coverage against resistant pathogens.

## Author contributions

Methodology: A. A. A., D. A., A. E. A., I. M. I., S. M. A., A. A. S. Investigation: M. E. E., F. M. K. A., A. M. E., T. A. Y., M. N. G., formal analysis and visualization: M. N. G., T. A. Y., M. E. E., S. M. A., A. E. A., F. M. K. A., I. M. B., A. A. S. Data curation and validation: A. G., A. M. E., A. A. A., D. A. conceptualization; A. G., M. N. G., T. A. Y. Supervision; A. G., A. M. E. writing – original draft; S. M. A., A. E. A., M. N. G. writing – review & editing; A. G., F. M. K. A, A. A. S., I. M. I., D. A.

## Conflicts of interest

The authors report no conflicts of interest.

## Data Availability

The data can be obtained from the corresponding author upon request.
